# Fatty Acids Reverse the Supramolecular Chirality of Insulin Fibrils

**DOI:** 10.1021/acs.jpclett.3c01527

**Published:** 2023-07-27

**Authors:** Aidan P. Holman, Kimberly Quinn, Rakesh Kumar, Sebastian Kmiecik, Abid Ali, Dmitry Kurouski

**Affiliations:** † Department of Entomology, Texas A&M University, College Station, Texas 77843, United States; ‡ Department of Biochemistry and Biophysics, Texas A&M University, College Station, Texas 77843, United States; § BioTools, Inc., Jupiter, Florida 33478, United States; ∥ Biological and Chemical Research Center, University of Warsaw, Warsaw 02-089, Poland

## Abstract

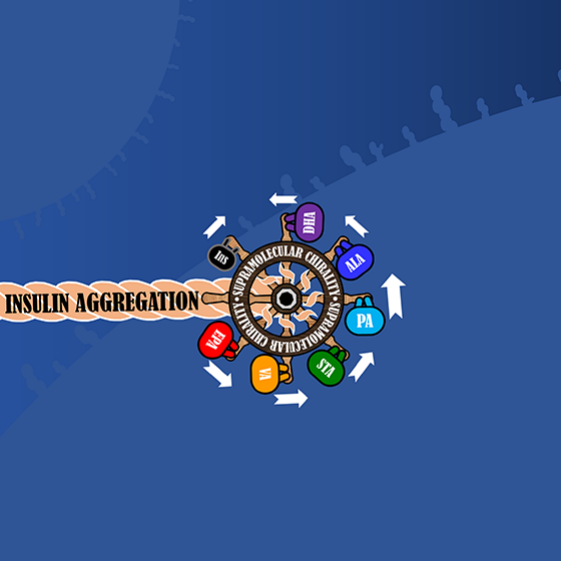

Long-chain unsaturated and polyunsaturated fatty acids (LCUFAs and LCPUFAs, respectively) are the essential components of phospholipids and sphingolipids, major building blocks of plasma and organelle membranes. These molecules are also involved in cell signaling and energy metabolism. Hence, both LCUFAs and LCPUFAs are broadly used as food supplements. However, the role of these fatty acids (FAs) in the self-assembly of misfolded proteins remains unclear. In this study, we investigated the effect of LCUFAs and LCPUFAs, as well as their saturated analogue, on insulin aggregation. Using vibrational circular dichroism, we found that all analyzed FAs reversed the supramolecular chirality of insulin fibrils. Molecular dynamics simulations showed that strong hydrophobic interactions were formed between the long aliphatic tails of FAs and hydrophobic amino acid residues of insulin. We infer that such insulin:FA complexes had different self-assembly mechanisms compared to that of insulin alone, which resulted in the observed reversal of the supramolecular chirality of the amyloid fibrils.

Phospho- and sphingolipids are the major constituents of plasma and organelle membranes.^1,2^ These lipids contain fatty acids (FAs) esterified to glycerol and sphingosine. The saturation and length of FAs in lipids determine their membrane fluidity.^3,4^ A rule of thumb is that with an increase in the number of double bonds, the melting points on lipids decrease, with drastic increases in membrane fluidity. An opposite relationship is found for the length of FAs in lipids. With an increase in the number of carbon atoms, the melting points of lipids increases.^1,2^ Consequently, the fluidity of membranes that possess such lipids decreases. These two physical parameters are constantly attenuated by living organisms to adjust the fluidity of their cell membranes in response to the constantly changing environment. FAs, including long-chain unsaturated and polyunsaturated fatty acids (LCUFAs and LCPUFAs, respectively), are also involved in the energy metabolism and growth of the retina, neurons, and skeletal muscle tissues, as well as cell signaling.^1,2^ This is why LCUFAs and LCPUFAs are broadly used as food supplies.

In the bloodstream and intercellular space, consumed LCUFAs and LCPUFAs interact with many molecules, including proteins and carbohydrates.^5,6^ Matveyenka and co-workers recently reported that such interactions significantly decreased the stability of insulin, a small hormone that regulates glucose metabolism.^7,8^ This triggers protein aggregation, which results in the formation of amyloid oligomers and fibrils, highly toxic species for surrounding cells. Furthermore, the presence of such aggregates in the bloodstream and intercellular space can trigger irreversible aggregation of other amyloidogenic proteins such as transthyretin and lysozyme, which ultimately leads to systemic amyloidosis, a severe disease that is characterized by a progressive accumulation of protein deposits in various organs and tissues.^9−12^

Rizevsky and co-workers recently reported that phospho- and sphingolipids could alter the supramolecular chirality of insulin and lysozyme fibrils if the lipids were present during protein aggregation.^13^ Furthermore, insulin and lysozyme fibrils with opposite supramolecular chirality exert drastically different cell toxicities on N27 rat dopaminergic cells.^13^ The question to ask is whether the observed effect of lipids is determined by the FAs present in such lipids. To answer this question, we used vibrational circular dichroism (VCD), a powerful spectroscopic technique that could be used to determine the absolute configuration of chiral molecules^14−19^ and to probe the supramolecular chirality of amyloid aggregates.^20−23^ For instance, Felippe and co-workers used VCD to unravel the absolute configuration and solution-state conformers of three peperomin-type secolignans isolated from *Peperomia blanda* (Piperaceae).^24^ Taniguchi and co-workers demonstrated that VCD was highly sensitive to various configurations of hydroxy fatty acids, lipid hydroperoxides, and lipid epoxides,^25^ as well as phospholipids.^26^

We also used atomic force microscopy (AFM) to investigate the extent to which various LCUFAs and LCPUFAs, as well as their saturated analogue, stearic acid (STA), could alter the supramolecular chirality of insulin fibrils. We also employed molecular dynamics (MD) simulations to investigate mechanisms of insulin:FA interactions. Our results indicate that the hydrophobic amino acids of insulin allow for hydrophobic interactions with the aliphatic tails of fatty acids. Such insulin:FA complexes have different mechanisms of self-assembly compared to that of insulin itself, which ultimately results in the formation of fibrils with opposite supramolecular chiralities.

First, we exposed solutions of bovine insulin that contained STA and various LCUFAs and LCPUFAs (Table 1) in 1:1 molar ratios at 37 °C for 48 h. In parallel, insulin, at the same concentration, was aggregated in the lipid-free environment (Ins). Next, we employed AFM to examine the morphology of protein aggregates formed under these experimental conditions. We found that in the lipid-free environment insulin aggregated in the form of thin fibrillar species that were 10–12 nm in height (Figure 1). These aggregates were 300–500 nm in length. Morphologically similar aggregates with the same lengths and heights were observed in other samples (Figure 1). Nevertheless, Ins:PA, Ins:ALA, and Ins:STA fibrils appeared to be thinner than the fibrils observed in other samples. These results demonstrated that after aggregation at 37 °C for 48 h, insulin formed morphologically similar, if not identical, aggregates in the presence of FAs compared to those observed upon insulin aggregation in the lipid-free environment.

**Figure 1:**
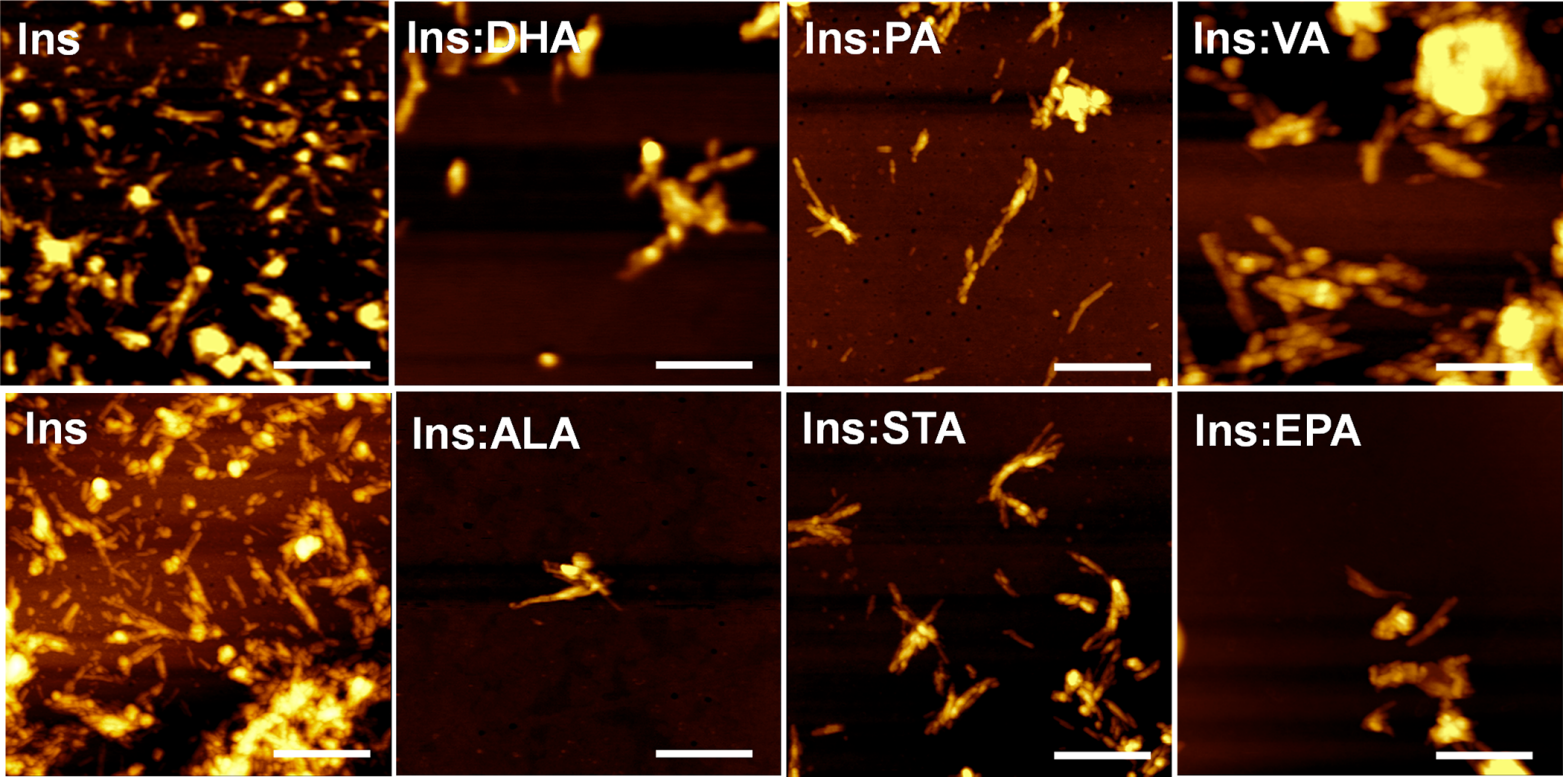
AFM-produced images of insulin aggregates formed in the lipid-free environment (Ins) and in the presence of LCUFAs and LCPUFAs. Scale bars are 500 nm.

**Table 1:** Names, Abbreviations, and Chemical Structures of FAs Used in Our Study

name	abbreviation	structure
docosahexaenoic acid	DHA	(4*Z*,7*Z*,10*Z*,13*Z*,16*Z*,19*Z*)-docosahexenoic acid
α-linolenic acid	ALA	(9*Z*,12*Z*,15*Z*)-α-linolenic acid
palmitoleic acid	PA	9-hexadecenoic acid
stearic acid	STA	octadecanoic acid
vaccenic acid	VA	(11*E*)-octadec-11-enoic acid
eicosapentaenoic acid	EPA	(5*Z*,8*Z*,11*Z*,14*Z*,17*Z*)-eicosapentenoic acid

We used VCD to examine the secondary structure and supramolecular chirality of these fibrils. We found that the VCD spectrum of Ins exhibited a negative peak at 1624 cm^–1^ and a positive peak at 1640 cm^–1^, as shown in Figure 2. The same VCD fingerprint was previously reported for insulin fibrils grown at pH 3.0 by the groups of Nafie and Lednev.^22,27−29^ Thus, our results are in good agreement with those for the previously reported fibrils.^13,23,29^ We also found that the VCD spectra acquired from Ins:DHA, Ins:ALA, Ins:PA, Ins:STA, Ins:VA, and Ins:EPA were nearly mirror images of the VCD spectrum of Ins. These results demonstrated that the presence of FAs at the stage of insulin aggregation reversed the supramolecular chirality of the insulin fibrils. We also found that the intensities of 1624 and 1640 cm^–1^ in the VCD spectra slightly varied between different protein samples (Figure 2). Ma and co-workers previously demonstrated that changes in the intensity of these vibrational bands could be used to track maturation of fibrillar species.^29^ Therefore, we can conclude that changes in the intensities of the 1624 and 1640 cm^–1^ bands indicate different degrees of maturation of insulin fibrils in the analyzed protein samples.

**Figure 2:**
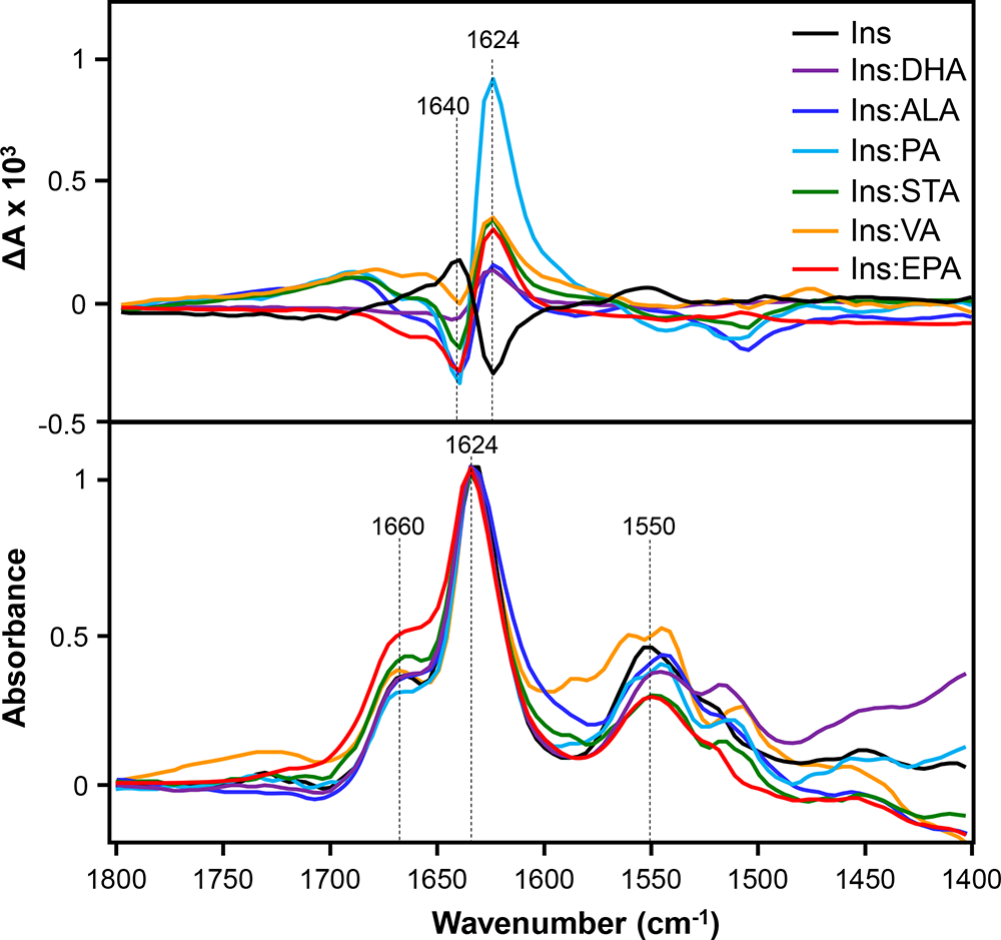
VCD (top) and IR (bottom) spectra of insulin fibrils formed in a lipid-free environment (Ins) and in the presence of LCUFAs and LCPUFAs. IR spectra were normalized at 1624 cm^–1^.

It should be noted that the IR spectra acquired from insulin fibrils formed in the lipid-free environment and in the presence of LCUFAs and LCPUFAs were very similar. In all acquired spectra, we observed an intense amide I centered around 1624 cm^–1^, which indicates the predominance of parallel β-sheet in the secondary structure of insulin fibrils.^29,30^ We also found a shoulder at 1660 cm^–1^, which could be assigned to the unordered secondary structure of all collected IR spectra of protein aggregates.^30^ On the basis of these results, we can conclude that the secondary structures of insulin fibrils grown in the lipid-free environment and in the presence of LCUFAs and LCPUFAs were very similar.

One can expect that the observed changes in the supramolecular chirality of insulin fibrils could be attributed to hydrophobic and hydrophilic interactions that could take place between insulin and FAs. Such protein:FA complexes would likely have different mechanisms of self-assembly compared to that of the protein alone, which will ultimately change the fold and, as a result, the supramolecular chirality of their fibrils. Therefore, we used molecular docking to determine binding affinities of ALA, DHA, EPA, PA, SA, and VA for insulin. We found the docking scores of insulin:FA complexes ranged from −5.3 to −7.3 kcal/mol (Table S1). These results indicated that insulin strongly interacted with all FAs. Furthermore, docking results indicate that our insulin:FA complexes were stabilized by two to three hydrogen and seven to eight hydrophobic bonds (Figure 3 and Figure S1).

**Figure 3:**
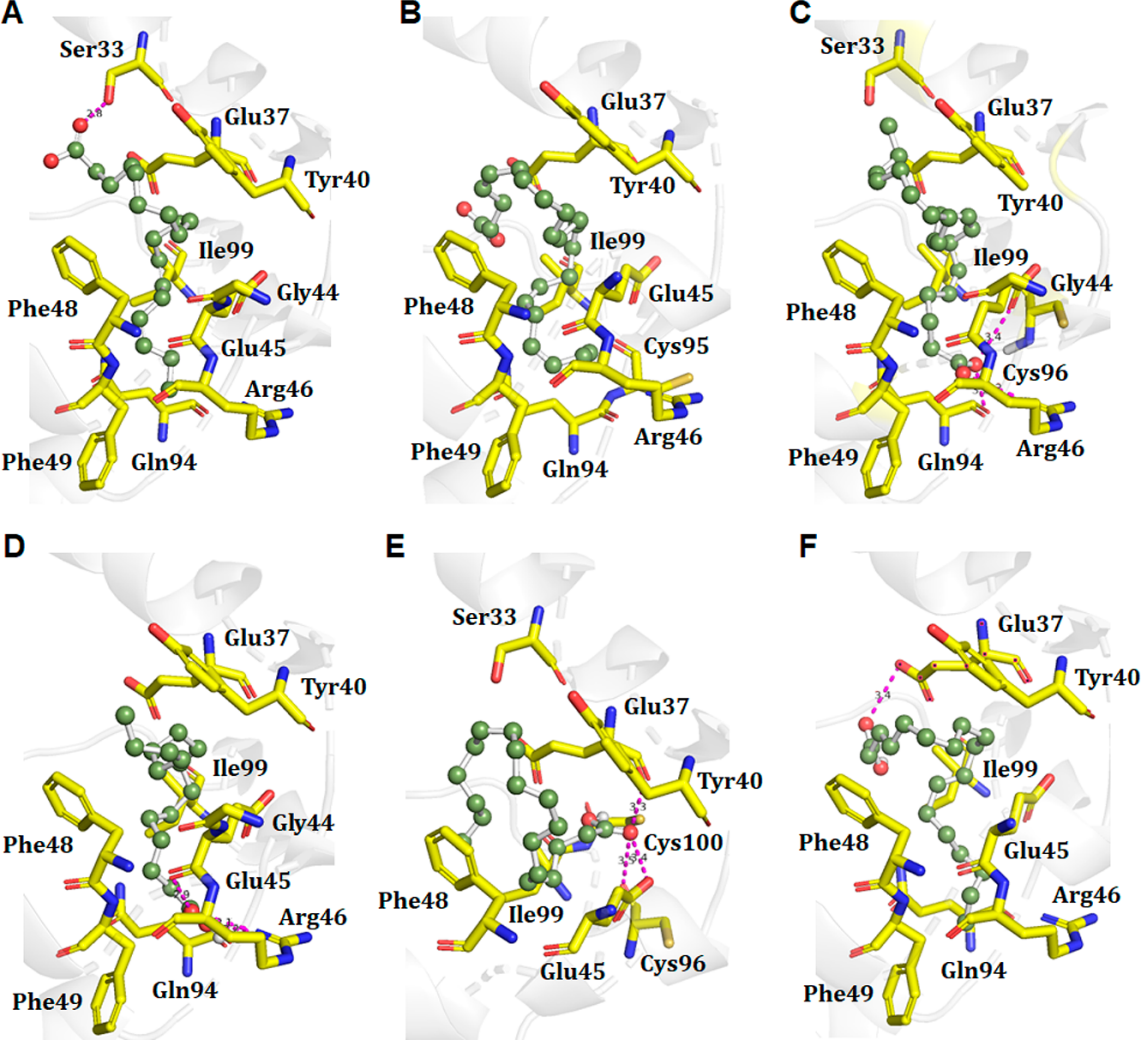
Three-dimensional plots of hydrophobic and hydrophilic interactions that take place between insulin and FAs: (A) Ins:ALA, (B) Ins:DHA, (C) Ins:EPA, (D) Ins:PA, (E) Ins:STA, and (F) Ins:VA. Interacting residues of proteins are shown as yellow sticks, while FAs are shown in ball-and-stick mode with a uniform green color. H-Bonds are shown as magenta dotted lines.

We also performed MD simulations on a 100 ns time scale for insulin and Ins:ALA, Ins:DHA, Ins:EPA, Ins:PA, Ins:SA, and Ins:VA. Root-mean-square deviations (RMSDs) for all models showed steady behaviors and convergence of the protein RMSD after 40 ns (Figure S2). Flexibilities of insulin in the absence and presence of FAs were analyzed by measuring the root-mean-square fluctuations (RMSFs). We found that the overall RMSFs were lower for insulin:FA complexes than for insulin itself (Figure S2). We also determined the compactness of insulin and insulin:FA complexes through the radius of gyration (*R*_g_). We found that *R*_g_ values were substantially smaller for insulin:FA complexes than for insulin alone. These results indicate that the binding of FAs to insulin changed the geometric parameters of the protein molecules. One may also expect that such geometric changes can alter the protein secondary structure of insulin. Our MD simulations showed that FA binding resulted in an increase in the helical content of insulin with a subsequent decrease in the amount of random coiling (Figure S3 and Table S2).

Our results showed that insulin strongly interacted with the polar heads and aliphatic tails of STA, LCUFAs, and LCPUFAs. Such interactions changed the molecular geometry, altering the secondary structure of insulin. As a result, insulin aggregated in the form of fibrils with the reversed supramolecular chirality compared to that of the aggregates grown in the lipid-free environment. These findings are significant because the results previously reported by Rizevsky and co-workers indicated that insulin fibrils with the opposite supramolecular chirality exerted drastically different cell toxicities.^13^

## Supporting Information Available

Materials and methods, Figures S1–S3, and Tables S1 and S2 (PDF)
Transparent Peer Review report available (PDF)
