# Supplementary material for: Fatty Acids Reverse the Supramolecular Chirality of Insulin Fibrils
Source: J Phys Chem Lett. 2023 Jul 27;14(30):6935–9. doi: 10.1021/acs.jpclett.3c01527 (PMC10863027; doi:10.1021/acs.jpclett.3c01527)
Supplement: Supplementary file 1 — jz3c01527_si_001.pdf [file jz3c01527_si_001.pdf]

Fatty Acids Reverse Supramolecular Chirality of Insulin Fibrils

Aidan P. Holman,[1,2] Kimberly Quinn,[3] Rakesh Kumar,[4] Sebastian Kmiecik,[4] Abid Ali,[2] and Dmitry Kurouski[2]*

1. Department of Entomology, Texas A&M University, College Station, Texas 77843, United States
2. Department of Biochemistry and Biophysics, Texas A&M University, College Station, Texas, 77843, United States
3. BioTools, Inc., Jupiter, FL 33478 United States
4. Biological and Chemical Research Center, University of Warsaw, Warsaw 02-089, Poland

Supplementary Information

Materials and Methods:

Materials: Bovine insulin was purchased from Sigma-Aldrich (St. Louis, MO, USA). We also used EPA (Eicosapentaenoic acid, 5Z,8Z,11Z,14Z,17Z-eicosapentenoic acid, Calbiochem Cat: 324875-25MG), DHA (Docosahexaenoic acid, 4Z,7Z,10Z,13Z,16Z,19Z-docosahxenoic acid, Acros Organics CAS: 6217-54-5), ALA (alpha-Linolenic acid, 9Z,12Z,15Z-alpha-linolenic acid, Acros Organics CAS: 463-40-1), STA (Stearic acid, Octadecanoic acid, Ward's Science+ CAS: 57-11-4), VA (Vaccenic acid (*trans*), (11*E*)-Octadec-11-enoic acid, Indofine Chemical Company CAS: 506-17-2), and PA (Palmitoleic acid, 9-hexadecenoic acid, MP Biomedicals CAS: 373-49-9).

Fatty Acid Stock Preparation: Each fatty acid was weighed/aliquoted and dissolved/diluted to 40 mM using PB. Diluted samples were placed in an ultrasonic water bath for 30-60 min (50 °C). Samples were periodically vortexed every 5-10 min. Once the FAs were dissolved, samples were diluted to a working concentration of 400 μM using PB. 40 mM stocks were stored in -20 ℃ at pH 7. We used 40 mM LCUFAs and LCPUFAs in all experiments.

Insulin aggregation: In the FA-free environment, 200 μM of insulin were dissolved in PBS. After that, the pH of the protein solution was adjusted to 3.0 using concentrated HCl. For LCUFA- and LCPUFA-samples, insulin (200 μM) was mixed with an equivalent concentration of the corresponding LCUFAs and LCPUFAs (200 μM). Next, we adjusted pH of the final solution to 3.0 using concentrated HCl. Finally, protein samples were placed into a 96 well-plate and kept in a plate reader (Tecan, Männedorf, Switzerland) at 37 °C for 28 h under 510 rpm agitation.

Vibrational circular dichroism (VCD): VCD and Infrared (IR) spectra were acquired at BioTools, Inc, Jupiter, FL on a dual-source, DualPEM ChiralIR-2X Fourier transform VCD spectrometer equipped with a MCT detector. Prior to the VCD and IR analysis, samples were centrifuged at 20,000 g for 20-30 min to concentrate protein aggregates. Next, an aliquot of formed pellet in the sample was placed into CaF2 cell that was placed into DualPEM ChiralIR-2X. Spectral acquisition time was 4-5 h. The spectra were processed using GRAMS/AI 7.0 (Thermo Galactic, Salem, NH). IR spectra were normalized on 1624 cm^{-1}.

AFM imaging: The AFM analysis of the protein aggregates was performed using soft tapping-mode AFM micro-cantilevers, purchased from OPUS (Bulgaria, EU) with a 2.0 N/m force constant, 70 kHz

frequency, and 240 µm length on AIST-NT-HORIBA system (Edison, NJ). Analysis of collected images was performed using AIST-NT software (Edison, NJ).

Protein modelling and docking: The tertiary structure of insulin was modelled through Modeller10.3 tool.[1] Available PDB (protein data bank) structures had missing amino acids, therefore we constructed full length proteins through multiple templates of Modeller as described previously.[2] Before presenting the protein structure for further experiments, the structure was energy-minimized in GROMACS (Groningen machine for chemical simulation)[3] and followed for structure quality inspection with various tools. The structures of 6 fatty acid ligands such as ALA (PubChem ID: 132426821), DHA (PubChem ID: 445580), EPA (PubChem ID: 5282847), PA (PubChem ID: 985), SA (PubChem ID: 5281) and VA (PubChem ID: 5281127) were taken from the PubChem database (https://pubchem.ncbi.nlm.nih.gov/) in a 2D format and converted to 3D conformers using the Open Babel tool.[4]

Molecular docking was accomplished in Smina software.[5] Precisely, the receptor and ligand molecules were prepared using AutoDock tools by assigning the charges and the addition of a polar hydrogen to the receptor and Gasteiger charges to the ligand (fatty acids).[6] The binding site on the receptor was achieved by preparing a grid box with the appropriate size and dimensions to cover the entire receptor molecule. Docking was performed through Smina with around 80 Monte-Carlo iterations and 5 docking poses for each ligand molecule that was generated. Binding affinities were measured in kilo calorie per mole (Kcal/mol) and the top protein-ligand docking complexes were analysed. 2D and 3D plots were generated to analyze the hydrophobic and hydrophilic interactions between protein and ligand molecules which was achieved through LigPlot+ (Version 2.2.8) and PyMOL (The PyMOL Molecular Graphics System, Version 1.3 Schrodinger, LLC) programs, respectively.[7]

Molecular dynamics simulation and binding free energy calculations: The GROMACS simulation package was used for molecular dynamics (MD) simulation for apo and complex proteins as described previously.[2-3] Briefly, protein and ligand topologies were derived from the GROMACS and SwissParam servers, respectively, using CHARMM force field.[8] Thereafter, both apo and complex proteins were immersed in water in triclinic boxes with SPC/E water model of defined periodic boundary conditions. All systems were neutralised with the addition of Na^+ and Cl^- ions followed by energy minimization. After that, two equilibration steps were followed: initially, the temperature of 300K for 500ps and pressure of 1bar for 1000ps were maintained by V-rescale thermostat and Parrinello-Rahman barostat, respectively. Finally, all systems including apo (Insulin) and complexes with 6 fatty acid ligands (ALA, DHA, EPA, PA, SA and VA) were conducted for 100ns in which 2fs time steps were applied. Trajectory analyses were done using the in-built tools of GROMACS. Binding free energies were computed by MM-PBSA (Molecular mechanics-Poisson-Boltzmann surface area) method using g_mmpbsa script.[9]

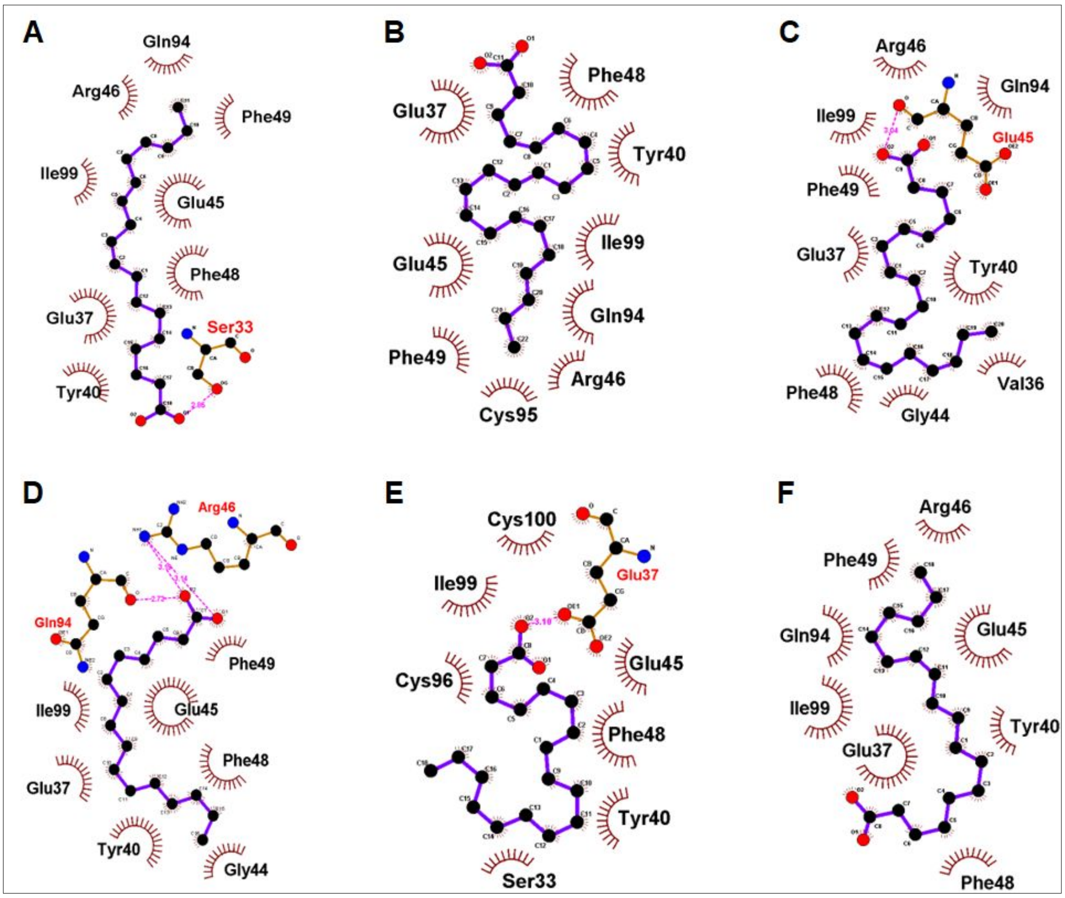

Figure S1. 2D plots of insulin:FAs interactions. (A) Ins:ALA, (B) Ins:DHA, (C) Ins:EPA, (D) Ins:PA, (E) Ins:STA and (F) Ins:VA. Hydrophobic interactions are indicated by red arc towards the ligand atoms they contact. Hydrophilic interactions are shown magenta colour.

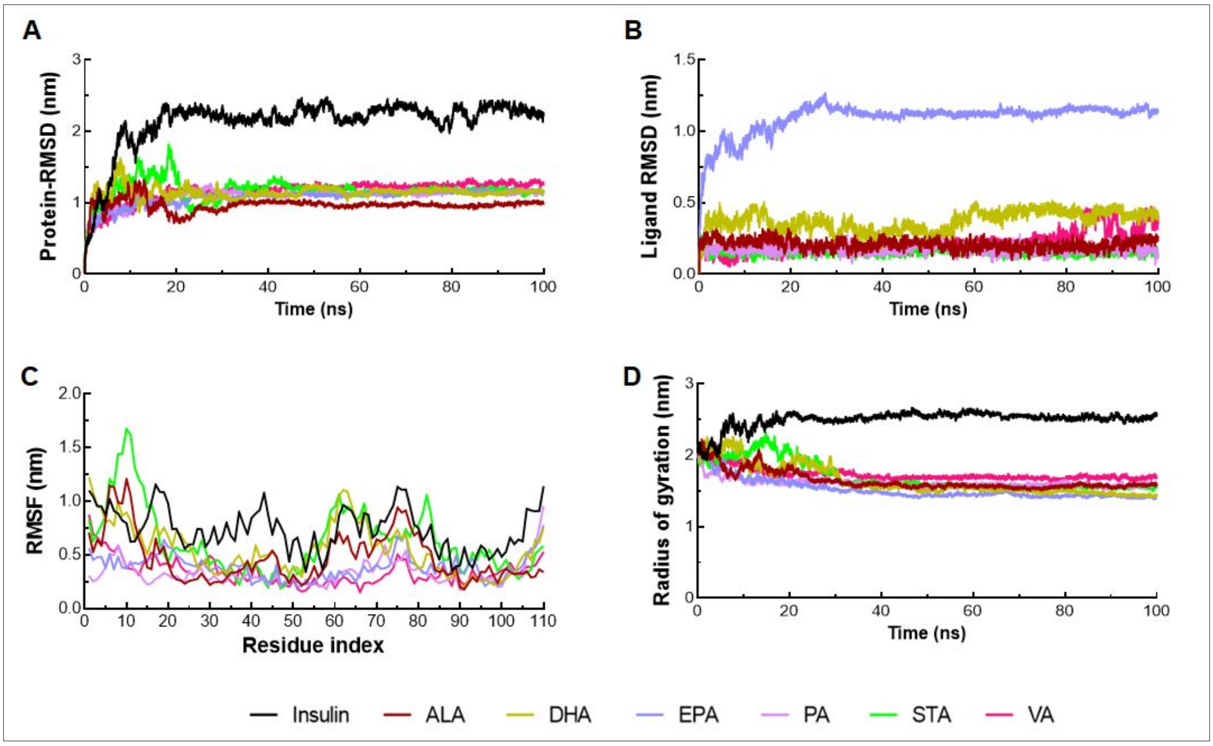

Figure S2. RMSD, RMSF and Rg profiles of insulin and insulin:FAs complexes. (A) Insulin RMSD at y-axis along the time in nanosecond at x-axis, (B) FAs RMSD at y-axis along the time in nanosecond at

x-axis, (C) RMSF of protein at y-axis with respect to residues at x-axis, and (D) Rg of protein at y-axis along the time in nanosecond at x-axis. Insulin and insulin:FAs complexes with ALA, DHA, EPA, PA, STA and VA are shown in black, red, mustard, light blue, magenta, green and pink colour lines, respectively.

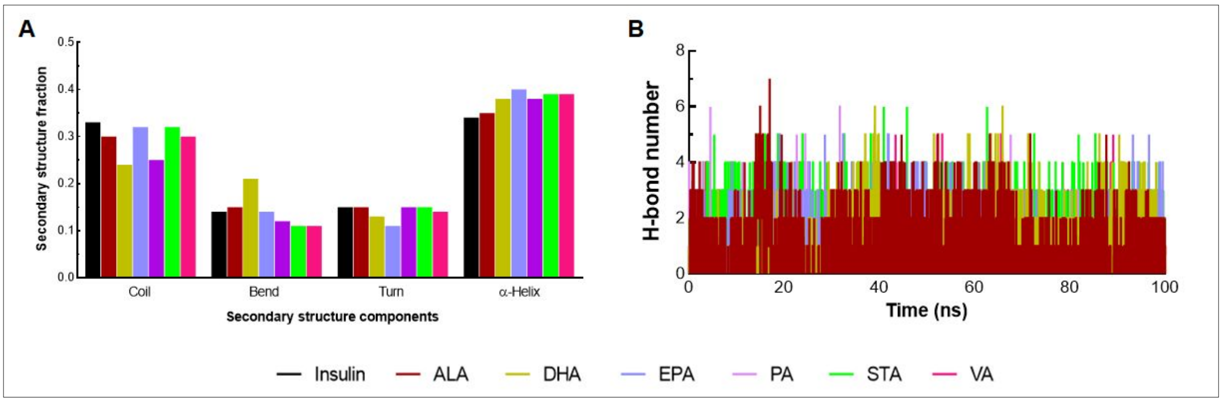

Figure S3. Structural properties analyses. (A) Secondary structures formation throughout the simulation period and (B) hydrogen bond number at y-axis along the time frame at x-axis throughout the simulation time. Insulin and insulin:FAs complexes with ALA, DHA, EPA, PA, STA and VA are shown in black, red, mustard, light blue, magenta, green and pink colour lines, respectively.

Table S1. Docking of 6 ligands (Binding affinities are measured in kcal/mol)

No	Ligand name	Docking scores
1	ALA	-6.0
2	DHA	-7.3
3	EPA	-6.9
4	PA	-5.7
5	SA	-5.3
6	VA	-5.7

Table S2. Secondary structure fractions

	Coil	Bend	Turn	α-Helix
**Insulin**	0.33	0.14	0.15	0.34
**ALA**	0.3	0.15	0.15	0.35
**DHA**	0.24	0.21	0.13	0.38
**EPA**	0.32	0.14	0.11	0.4
**PA**	0.25	0.12	0.15	0.38
**SA**	0.32	0.11	0.15	0.39
**VA**	0.3	0.11	0.14	0.39

References:

1. Sali, A.; Overington, J. P., Derivation of Rules for Comparative Protein Modeling from a Database of Protein Structure Alignments. *Protein Sci* **1994**, *3*, 1582-96.
2. Kumar, R.; Maurya, R.; Saran, S., Identification of Novel Inhibitors of the Translationally Controlled Tumor Protein (Tctp): Insights from Molecular Dynamics. *Mol Biosyst* **2017**, *13*, 510-524.
3. Van Der Spoel, D.; Lindahl, E.; Hess, B.; Groenhof, G.; Mark, A. E.; Berendsen, H. J., Gromacs: Fast, Flexible, and Free. *J Comput Chem* **2005**, *26*, 1701-18.

4. O'Boyle, N. M.; Banck, M.; James, C. A.; Morley, C.; Vandermeersch, T.; Hutchison, G. R., Open Babel: An Open Chemical Toolbox. *J Cheminform* **2011**, *3*, 33.
5. Koes, D. R.; Baumgartner, M. P.; Camacho, C. J., Lessons Learned in Empirical Scoring with Smina from the Csar 2011 Benchmarking Exercise. *J Chem Inf Model* **2013**, *53*, 1893-904.
6. Kumar, R.; Saran, S., Structure, Molecular Dynamics Simulation, and Docking Studies of Dictyostelium Discoideum and Human Straps. *J Cell Biochem* **2018**, *119*, 7177-7191.
7. Laskowski, R. A.; Swindells, M. B., Ligplot+: Multiple Ligand-Protein Interaction Diagrams for Drug Discovery. *J Chem Inf Model* **2011**, *51*, 2778-86.
8. Zoete, V.; Cuendet, M. A.; Grosdidier, A.; Michielin, O., Swissparam: A Fast Force Field Generation Tool for Small Organic Molecules. *J Comput Chem* **2011**, *32*, 2359-68.
9. Kumari, R.; Kumar, R.; Open Source Drug Discovery, C.; Lynn, A., G_Mmpbsa--a Gromacs Tool for High-Throughput Mm-Pbsa Calculations. *J Chem Inf Model* **2014**, *54*, 1951-62.
