# Supplementary material for: Fatty Acids Reverse the Supramolecular Chirality of Insulin Fibrils
Source: J Phys Chem Lett. 2023 Jul 27;14(30):6935–9. doi: 10.1021/acs.jpclett.3c01527 (PMC10863027; doi:10.1021/acs.jpclett.3c01527)
Supplement: Supplementary file 2 — jz3c01527_si_002.pdf [file jz3c01527_si_002.pdf]

jz-2023-015275.R1

Name: Peer Review Information for "Fatty Acids Reverse Supramolecular Chirality of Insulin Fibrils"

First Round of Reviewer Comments

Reviewer: 1
Comments to the Author
Kurouski and co-workers present a fibril aggregation study focussing on insulin and its interaction with different fatty acids. They demonstrate that fatty acids lead to a chag ein aggregation behaviour as deduced from a change in the VCD spectra of the fibrils. I cannot find an major issue with the manuscript, besides the fact that I still have doubts about the general way fibril-VCD is analysed. It seems accepted in the community that sign changes in the spectra refer to changes in the fibril superstructure, but I have a constant urge to oppose as I fear these could be artefacts from birefringence. But putting this aside, as mentioned, it seems commonly accepted in the VCD community, there are still some points to be addressed:

1) There is no information in the manuscript on the VCD measurements. Seems they were done by BioTools Inc, but nonetheless some experimental detailed would be required. Have the samples been rotated during the measurement? Have the sampled been turned around (the x-axis) as proposed for solid state samples showing birefringence artefacts? Have samples been measured in dried state or in aqueous solution/suspension? Measurement times and other conditions?

2) The absorbance of the samples is very close to 1.0, while it is typically recommended to stay below 0.9 to avoid artefacts. Please comment on this and explain why the measurements should not be re-done at lower concentrations/absorbance values.

3) The spectra have many more features, can they be assigned to anything? Do the spectra provide any spectroscopic indications or marker bands that confirm the incorporation of the fatty acids into the structure?

4) The AFM-pictures are fairly small and not particularly sharp (low resolution). The should be enlarged and complemented with additional figures in the SI. Comparing the figures among each other, I find it intersting that the author comment only on the height but not on the lengths of the fibrils, especially as this seems to be important for the argumentation in the interpretation of the VCD (different maturation states...).

5) Likewise, the AFM-pictures do not allow my to conclude that "morphologically similar" fibrils are formed. For a layperson in AFM, which I am, the pictures if Ins and Ins:PA look fairly different...

6) If the VCD spectra show different states of maturation, couldn't this be shown also be AFM?

7) Figure 3 is very low res and cannot be read. From the 2D-plot in Figure S1 it seems that the binding side of the FA is always the same. Hence, it would make sense to me to highlight this more, especially by showing alle the figures in the same orientation, so that one does not hav to read all AA labels to figure it out by oneself. Then it also becomes apparent that the FA do not fold in exactly the same way (which could be worth commenting on, too).

8) In the docking and MD analysis, I am missing error bars for the given estimates. Have the calculations been done from different starting points? I change in helix content by 4% for a single run would otherwise be something I would not want to put too much emphasis on...

9) "Our results also showed that fibril supramolecular chirality could not be revealed by the microscopic analysis of such aggregates." Unless I missed something, the present story also does not say much about the supramolecular chirality besides saying that there is fibrilation and that the VCD spectra indicate different types of aggregation.

Reviewer: 2
Comments to the Author
In the manuscript “Fatty Acids Reverse Supramolecular Chirality of Insulin Fibrils”, the authors use vibrational circular dichroism (VCD) together with molecular dynamics simulation to study the effect of fatty acids on insulin aggregation. The main conclusion of the work is that long-chain fatty acids can reverse the chirality of the aggregates, as suggested by the title. However, the experimental conditions are not presented clearly, and more experiments seem to be needed to reach the above conclusion. The Methods section also requires a more detailed description so other groups can reproduce the results. Therefore, I cannot recommend publishing this manuscript in the current form.
The issues that should be addressed:
1. There is no information about the VCD experiments under the Method section. What instrument is used? What are the measurement conditions? Is the VCD measurement performed in liquid or on the dried sample? The MD simulation included the water model, so the measurements should be performed under liquid conditions for a comparable result.
2. In order to conclude the effect of fatty acids, only measuring the sample with/without fatty acids is insufficient. A concentration dependence study must be performed. This will show if the change in chirality correlates with the fatty acids added. A positive correlation is the first step. However, as we all know, correlation does not mean causation.
3. It is not clear about the amount of fatty acid added to the insulin. The authors only stated that they use equal concentrations of STA, fatty acids, and insulin. Adding 100 uL of 400 uM of solution A into 100 uL of 400 uM solution B is a very different system compared to 500 uL of A in 100 uL of B.
4. Many other factors can affect insulin aggregation. For example, the authors studied the pH effect in a previous Chem Comm (2010) publication. A pH dependence study with the fatty acid added will be a sensible experiment to examine if there is any cross-correlation between pH and fatty acids.

5. Vibrational spectroscopy is sensitive to intermolecular interactions and can be simulated from a proposed structure. I wonder what the simulated spectra will be if using the structures revealed by the MD simulation. A whole system simulation may not be possible due to the computation cost. However, since the authors identified the interacting chemical groups, how good are the simulated spectra on those groups agreeing with the experimental results? Typically, X-Ray techniques (SAXS) are suited for structural analysis. Vibrational spectroscopy (VCD and IR) can provide information, but more analysis needs to be done.
6. The authors should be cautious with the excess amount of self-citation. Eleven out of eighteen (60%) is too much. This is a vibrant research topic, which ties with the potential impact of this work. A more inclusive and relevant list of references should be presented.
Minor Issues:
Figure 3. The figure resolution is too low to distinguish the labels clearly. I try to use the ones in the supporting document (which have the proper resolution) to understand this figure. White sticks on a white background provide another challenge to see the suggested interactions.

Author's Response to Peer Review Comments:
Please find point-by-point response attached

1) TOC Graphic: Please resize the TOC graphic per journal guidelines (2 in x 2 in).

Response: addressed

2) Graphics: Please improve the quality of Graphic 3.

Response: addressed

3) Headers: Remove the section heading(s) throughout the body of the manuscript (you can leave Methods and Abstract headings).

Response: addressed

4) References: In both the main file and the supporting information, fix the style of all references to use JPCL formatting (check all references carefully). ***JPC Letters reference formatting requires that journal references should contain: () around numbers, author names, article title (titles entirely in title case or entirely in lower case), abbreviated journal title (italicized), year (bolded), volume (italicized), and pages (first-last). Book references should contain author names, book title (in the same pattern), publisher, city, and year. Websites must include date of access.

Response: addressed

5) Supporting Information: Please number SI pages in the following format: “S1, S2…”

Response: addressed

Reviewer: 1

1) There is no information in the manuscript on the VCD measurements. Seems they were done by BioTools Inc, but nonetheless some experimental detailed would be required. Have the samples been rotated during the measurement? Have the sampled been turned around (the x-axis) as proposed for solid state samples showing birefringence artefacts? Have samples been measured in dried state or in aqueous solution/suspension? Measurement times and other conditions?

Response: we apologize for missing the information about VCD measurements. VCD and Infrared (IR) spectra were measured at BioTools, Inc, Jupiter, FL using a dual-source, DualPEM ChiralIR-2X Fourier transform VCD spectrometer equipped with an MCT detector. Prior to the VCD and IR analysis, samples were centrifuged at 20,000 g for 20-30 min to concentrate protein aggregates. Next, an aliquot of formed pellet in the sample was placed into CaF_2 cell that was placed into DualPEM ChiralIR-2X. Spectral acquisition time was 4-5 h.

We have not rotated (turned around) the samples because the samples were measured in solution. We have reproducibly observed positive and negative VCD signals from insulin fibrils formed in the FA-free and FA-rich environments. It should be noted that polyglutamine fibrils (polyQ) provide completely different VCD spectra if measured under the same experimental conditions (doi: 10.1016/j.febslet.2013.03.038). Thus, we are convinced that VCD measures the supramolecular chirality rather than artifacts.

To ensure the absence of birefringence artefacts in the reported IR/VCD spectra, we rotated the CaF2 cell to 45 ° and 90 °. The same VCD/IR spectra were observed compared to those acquired at 0 °.

To address the reviewer’s suggestion, we added the following description of VCD to the materials and methods of the manuscript.

Vibrational circular dichroism (VCD): VCD and Infrared (IR) spectra were acquired at BioTools, Inc, Jupiter, FL on a dual-source, DualPEM ChiralIR-2X Fourier transform VCD spectrometer equipped with a MCT detector. Prior to the VCD and IR analysis, samples were centrifuged at 20,000 g for 20-30 min to concentrate protein aggregates. Next, an aliquot of formed pellet in the sample was placed into CaF_2 cell that was placed into DualPEM ChiralIR-2X. Spectral acquisition time was 4-5 h. The spectra were processed using GRAMS/AI 7.0 (Thermo Galactic, Salem, NH).

2) The absorbance of the samples is very close to 1.0, while it is typically recommended to stay below 0.9 to avoid artefacts. Please comment on this and explain why the measurements should not be re-done at lower concentrations/absorbance values.

Response: we apologize for the possible misinterpretation of the reported results. Absorbance in all analyzed samples was within 0.2-0.4. However, we normalized the reported spectra at 1624 cm^{-1}.

To address the reviewer’s suggestion, we added the following sentence to the materials and methods of the manuscript, as well las to the legend of the Figure 2.

“IR spectra were normalized on 1624 cm^{-1}.”

3) The spectra have many more features, can they be assigned to anything? Do the spectra provide any spectroscopic indications or marker bands that confirm the incorporation of the fatty acids into the structure?

Response: Extensive computational simulations are required to fully understand all features in the VCD spectra. We only assigned and interpreted the vibrational bands which origin was elucidated by Schweitzer-Stenner’s group (DOI: 10.1021/ja1089827) and confirmed by Nafie group for a large number of amyloid aggregates (doi: 10.1016/j.bpj.2012.04.042; doi: 10.1021/ja407583r).

4) The AFM-pictures are fairly small and not particularly sharp (low resolution). The should be enlarged and complemented with additional figures in the SI. Comparing the figures among each other, I find it interesting that the author comment only on the height but not on the lengths of the fibrils, especially as this seems to be important for the argumentation in the interpretation of the VCD (different maturation states...).

Response: we enlarged the picture in the revised manuscript. We also compared the length of fibrils and found that fibrils in all analyzed samples had similar lengths (300-500 nm).

To address the reviewer’s suggestion, we modified the following sentences in the manuscript: “These aggregates had 300-500 nm in length. Morphologically similar aggregates with the same lengths and heights were observed in other samples, Figure 1.”

5) Likewise, the AFM-pictures do not allow my to conclude that "morphologically similar" fibrils are formed. For a layperson in AFM, which I am, the pictures if Ins and Ins:PA look fairly different...

Response: we agree with the observation made by the reviewer. To address the reviewer's suggestion, we added the following sentence to the manuscript:

"Nevertheless, Ins:PA, Ins:ALA, and Ins:STA fibrils appeared to be thinner than fibrils observed in other samples."

6) If the VCD spectra show different states of maturation, couldn't this be shown also be AFM?

Response: We agree that such a correlation study should be interesting. Similar results were shown for insulin (doi: 10.1016/j.bpj.2012.04.042) by Nafie group. It was also shown that the magnitude of VCD spectra increase as the time of protein aggregation increases (doi: 10.1021/ja074188z). We think that VCD/AFM analysis of maturation of insulin fibrils formed in the presence of FAs can be the subject of our future study.

7) Figure 3 is very low res and cannot be read. From the 2D-plot in Figure S1 it seems that the binding side of the FA is always the same. Hence, it would make sense to me to highlight this more, especially by showing all the figures in the same orientation, so that one does not hav to read all AA labels to figure it out by oneself. Then it also becomes apparent that the FA do not fold in exactly the same way (which could be worth commenting on, too).

Response: We have improved the resolution and layout of Figure 3 for easy understating. Further, all protein-ligand interacting complexes are displayed in the same orientations shown in figure3 as suggested. We are unable to do such changes in 2d plots due to tool (LigPlot) limitation. However, we used a simple plot for easy understanding in figure S1.

8) In the docking and MD analysis, I am missing error bars for the given estimates. Have the calculations been done from different starting points? I change in helix content by 4% for a single run would otherwise be something I would not want to put too much emphasis on...

Response: Thank you for the comment and you are right that there are no error bars in docking and MD calculations. We ran single docking with exhaustiveness (Monte-Carlo iteration) ranging from 40-50 to saturate the ligand binding search space as thoroughly as possible. It was found that a single run with range of 80 exhaustiveness is considered sufficient for most of applications. For MD simulations, out of 5 we selected top scored docking binding pose based on the minimum energy with maximum binding affinity for each protein-ligand complex.

We agree with reviewer that we found 4% increase in helix content in case of all ligands except ALA (minor increase) as compared to apo protein during single MD run. However, we are emphasizing this because we performed this analysis on stabilized MD trajectories. We will get similar results if we repeat the simulation or done in duplicate.

9) "Our results also showed that fibril supramolecular chirality could not be revealed by the microscopic analysis of such aggregates." Unless I missed something, the present story also does not say much about the supramolecular chirality besides saying that there is fibrilation and that the VCD spectra indicate different types of aggregation.

Response: we agree with this suggestion. We removed this sentence from the revised manuscript.

Reviewer: 2

1. There is no information about the VCD experiments under the Method section. What instrument is used? What are the measurement conditions? Is the VCD measurement performed in liquid or on the dried sample? The MD simulation included the water model, so the measurements should be performed under liquid conditions for a comparable result.

Response: we apologize for missing the information about VCD measurements. VCD and Infrared (IR) spectra were measured at BioTools, Inc, Jupiter, FL using a dual-source, DualPEM ChiralIR-2X Fourier transform VCD spectrometer equipped with an MCT detector. Prior to the VCD and IR analysis, samples were centrifuged at 20,000 g for 20-30 min to concentrate protein aggregates. Next, an aliquot of formed pellet in the sample was placed into CaF_2 cell that was placed into DualPEM ChiralIR-2X. Spectral acquisition time was 4-5 h.

We have not rotated (turned around) the samples because the samples were measured in solution. We have reproducibly observed positive and negative VCD signals from insulin fibrils formed in the FA-free and FA-rich environments. It should be noted that polyglutamine fibrils (polyQ) provide completely different VCD spectra if measured under the same experimental conditions (doi: 10.1016/j.febslet.2013.03.038). Thus, we are convinced that VCD measures the supramolecular chirality rather than artifacts.

To ensure the absence of birefringence artefacts in the reported IR/VCD spectra, we rotated the CaF2 cell to 45 ° and 90 °. The same VCD/IR spectra were observed compared to those acquired at 0 °.

To address the reviewer's suggestion, we added the following description of VCD to the materials and methods of the manuscript.

Vibrational circular dichroism (VCD): VCD and Infrared (IR) spectra were acquired at BioTools, Inc, Jupiter, FL on a dual-source, DualPEM ChiralIR-2X Fourier transform VCD spectrometer equipped with a MCT detector. Prior to the VCD and IR analysis, samples were centrifuged at 20,000 g for 20-30 min to concentrate protein aggregates. Next, an aliquot of formed pellet in the sample was placed into CaF_2 cell that was placed into DualPEM ChiralIR-2X. Spectral acquisition time was 4-5 h. The spectra were processed using GRAMS/AI 7.0 (Thermo Galactic, Salem, NH).

2. In order to conclude the effect of fatty acids, only measuring the sample with/without fatty acids is insufficient. A concentration dependence study must be performed. This will show if the change in chirality correlates with the fatty acids added. A positive correlation is the first step. However, as we all know, correlation does not mean causation.

Response: In our previous study, we showed that an increase in the concentration of phospholipids relative to the concentration of lysozyme to 10:1 and 5:1 resulted in the increase in the rate of protein aggregation. However, no significant effect on the structure of the formed fibrils and their toxicity was observed (doi.org/10.1016/j.bbalip.2023.159305). One can expect that similar observations may be expected for FAs. Nevertheless, we completely agree with the reviewer that this is an important experiment that should be performed to fully understand the role of fatty acids on insulin aggregation. This experiment, however, is the subject for the separate study.

3. It is not clear about the amount of fatty acid added to the insulin. The authors only stated that they use equal concentrations of STA, fatty acids, and insulin. Adding 100 uL of 400 uM of solution A into 100 uL of 400 uM solution B is a very different system compared to 500 uL of A in 100 uL of B.

Response: we apologize for this confusion. The final concentration of insulin in all samples was 200 μM. The final concentration of all FAs in the samples was 200 μM.

To address the reviewer's suggestion, we modified the following sentences in the material and methods section of the manuscript:

"In the FA-free environment, 200 μM of insulin were dissolved in PBS. After that, the pH of the protein solution was adjusted to 3.0 using concentrated HCl. For LCUFA- and LCPUFA-samples, insulin (200 μM) was mixed with an equivalent concentration of the corresponding LCUFAs and LCPUFAs (200 μM)."

4. Many other factors can affect insulin aggregation. For example, the authors studied the pH effect in a previous Chem Comm (2010) publication. A pH dependence study with the fatty acid added will be a sensible experiment to examine if there is any cross-correlation between pH and fatty acids.

Response: we agree that it is important to understand the role of pH and other factors in the VCD of insulin fibrils formed in the presence of FAs. These studies will be the subject of our future work.

5. Vibrational spectroscopy is sensitive to intermolecular interactions and can be simulated from a proposed structure. I wonder what the simulated spectra will be if using the structures revealed by the MD simulation. A whole system simulation may not be possible due to the computation cost. However, since the authors identified the interacting chemical groups, how good are the simulated spectra on those groups agreeing with the experimental results? Typically, X-Ray techniques (SAXS) are suited for structural analysis. Vibrational spectroscopy (VCD and IR) can provide information, but more analysis needs to be done.

Response: This is an excellent idea, however, we think with the possessed computational resources it cannot be implemented at this moment. VCD calculations that were performed by Schweitzer-Stenner's group (DOI: 10.1021/ja1089827) were made on a small peptide that would not represent interactions with FAs. Alternatively, calculations of VCD of insulin:FAs fibrils would require the assembly of many of such protein:lipid complexes into one fibrillar structure. Such calculations would be too costly/slow at the moment. Therefore, we focused only on the elucidation of the interactions between the protein and FAs. We aim to resolve the structure of insulin:FAs using cryo-EM, which is subject for the separate study.

6. The authors should be cautious with the excess amount of self-citation. Eleven out of eighteen (60%) is too much. This is a vibrant research topic, which ties with the potential impact of this work. A more inclusive and relevant list of references should be presented.

Response: we are grateful to the reviewer for the provided suggestion. We modified the citations in the paper to avoid the excess of self-citation.

7. Figure 3. The figure resolution is too low to distinguish the labels clearly. I try to use the ones in the supporting document (which have the proper resolution) to understand this figure. White sticks on a white background provide another challenge to see the suggested interactions.

Response: we improved the resolution of Figure 3.

jz-2023-015275.R1

Name: Peer Review Information for "Fatty Acids Reverse Supramolecular Chirality of Insulin Fibrils"

Second Round of Reviewer Comments

Reviewer: 1
Comments to the Author
The authors have addressed my comments, so it should be fine from my side (although I also see many good suggestions by the other reviewer, which the authors all want to keep for future studies...). I have two minor comments, tho:

- Regarding Q7 and the orientation of the protein in Figure 3: In this view, it does not look like a too similar binding anymore. Can we learn something about a potential mechanism from them? Why would such binding enfore more helix content?

- Regarding Q8 concerning the 4% increase in helix content: The authors reponded to my questions regarding the significance of the change in helix content, that they "will get similar results if we repeat the simulation or done in duplicate." I must say, I find the number itself already small and not particularly significant (given the structural effects that should arise from it), but I would also not be too confident that the number would be correctly reproduced.
